# Mechanisims of asthma and allergic disease – 1066. Ceruloplasmin oxidase activity in allergic asthma and allergic rhinitis

**DOI:** 10.1186/1939-4551-6-S1-P64

**Published:** 2013-04-23

**Authors:** Arzu Didem Yalcin, Saadet Gumuslu, Gizem Genc, Atil Bisgin, Mustafa Yildiz, Aysegul Kargi, Reginald M Gorczynski

**Affiliations:** 1 Internal Medicine, Allergy and Immunology, Education and Research Hospital, Turkey; 2 Akdeniz University, Turkey; 3 Cancer Institute, Sweden; 4 Antalya Education and Research Hospital, Turkey; 5 Department of Internal Medicine, Antalya Education and Research Hospital, Antalya, Turkey; 6 Division of Cellular & Molecular Biology, Toronto Hospital, University Health Network, Toronto, ON, Canada

## Background

The known functions of ceruloplasmin oxidase activity (COA) include copper transportation, iron metabolism, antioxidant defense and involvement in angiogenesis and coagulation. The role of ceruloplasmin oxidase involving the interaction of oxidant and anti-oxidant balance in allergic diseases is still unknown. It was previously reported that synthesis of ceruloplasmin was situmulated by interleukin-1 in normal and copper-deficient rat models concluding that ceruloplasmin is dependent for oxidase activity. Moreover, the copper ions had been suggested as an explanation for the sensitivity of asthmatic individuals by their biologic effects of inhaled particulate air pollution. In vivo experiments on finding the cytokines involved in acute-phase protein response showed that there are three major cytokines; interleukin 1-beta, 6 and TNF-alpha. Our study was designed to examine the changes in COAs in severe persistent asthma-allergic rhinitis, new diagnosed allergic asthma-allergic rhinitis and allergic rhinitis patients.

## Methods

The study included 20 age- and sex-matched healthy individuals as control group (group I); group II was including 15 newly diagnosed allergic asthma - allergic rhinitis; group III was including 15 patients with severe persistent asthma - allergic rhinitis and in the fourth group there were 20 patients with allergic rhinitis. Group III was divided in two groups, severe persistent asthma-allergic rhinitis who were pre- (III-A) and post-treated (III-B) with omalizumab. Group IV was divided to two groups, pre- (IV-A) and post-treatment (IV-B) with specific subcutaneous immunotherapy modalities. All the post-treatment measurements were 12 months after the therapy. All the patients were assessed by the skin prick test, high sensitive C-reactive protein (hs-CRP) and COA.

## Results

hs-CRP and COA levels were measured in all groups. There were significant differences between group I and groups III-A, III-B, IV-A and IV-B; group II and groups III-A, III-B, IV-A and IV-B; group III-A and groups III-B, IV-A and IV-B; group III-A and groups IV-A and IV-B; and group IV-A and IV-B. Interestingly, there was a correlation between the hs-CRP and COA levels in Group III-A.

## Conclusions

Our data suggest that hs-CRP and COA levels might be an indicator of an inflammation and important in revelation of patients with allergy related diseases, especially of asthma patients.

